# Comparative genomics and phylogenetic discordance of cultivated tomato and close wild relatives

**DOI:** 10.7717/peerj.793

**Published:** 2015-02-26

**Authors:** Susan R. Strickler, Aureliano Bombarely, Jesse D. Munkvold, Thomas York, Naama Menda, Gregory B. Martin, Lukas A. Mueller

**Affiliations:** 1 Boyce Thompson Institute for Plant Research, Ithaca, NY, USA; 2 Department of Horticulture, Virginia Polytechnic Institute and State University, Blacksburg, VA, USA; 3 Department of Plant Pathology and Plant-Microbe Biology, Cornell University, Ithaca, NY, USA

**Keywords:** Tomato, Phylogeny, Solanum, Genome, Incomplete lineage sorting, Introgression, Selection, Phylogeny

## Abstract

**Background.** Studies of ancestry are difficult in the tomato because it crosses with many wild relatives and species in the tomato clade that have diverged very recently. As a result, the phylogeny in relation to its closest relatives remains uncertain. By using the coding sequence from *Solanum lycopersicum*, *S. galapagense*, *S. pimpinellifolium*, *S. corneliomuelleri*, and *S. tuberosum* and the genomic sequence from *S. lycopersicum* ‘Heinz’, an heirloom line, *S. lycopersicum* ‘Yellow Pear’, and two of cultivated tomato’s closest relatives, *S. galapagense* and *S. pimpinellifolium*, we have aimed to resolve the phylogenies of these closely related species as well as identify phylogenetic discordance in the reference cultivated tomato.

**Results.** Divergence date estimates suggest that the divergence of *S. lycopersicum*, *S. galapagense*, and *S. pimpinellifolium* happened less than 0.5 MYA. Phylogenies based on 8,857 coding sequences support grouping of *S. lycopersicum* and *S. galapagense*, although two secondary trees are also highly represented. A total of 25 genes in our analysis had sites with evidence of positive selection along the *S. lycopersicum* lineage. Whole genome phylogenies showed that while incongruence is prevalent in genomic comparisons between these genotypes, likely as a result of introgression and incomplete lineage sorting, a primary phylogenetic history was strongly supported.

**Conclusions.** Based on analysis of these genotypes, *S. galapagense* appears to be closely related to *S. lycopersicum*, suggesting they had a common ancestor prior to the arrival of an *S. galapagense* ancestor to the Galápagos Islands, but after divergence of the sequenced *S. pimpinellifolium*. Genes showing selection along the *S. lycopersicum* lineage may be important in domestication or selection occurring post-domestication. Further analysis of intraspecific data in these species will help to establish the evolutionary history of cultivated tomato. The use of an heirloom line is helpful in deducing true phylogenetic information of *S. lycopersicum* and identifying regions of introgression from wild species.

## Background

Identifying and exploiting diversity present in wild tomato species has been crucial for the improvement of production traits in cultivated tomato (Grandillo et al., 2011). Useful traits, such as ease of harvest, shelf life, pathogen resistance, and abiotic stress tolerance have been introduced through introgressions from wild species. The wild tomatoes, along with the cultivated tomato, *Solanum lycopersicum*, comprise the 13 members of *Solanum* sect. *Lycopersicon*, and are native to western South America. All members of the clade can be crossed to cultivated tomato with varying degrees of ease (Grandillo et al., 2011) and breeding programs for cultivated tomato have widely utilized this property since the 1940s (Grandillo et al., 2011), allowing for the introgression of traits desirable in fruit production. Interestingly, most wild species are green-fruited except for three: *S. pimpinellifolium*, *S. galapagense*, and *S. cheesmaniae*. These species are also thought to be the closest relatives to cultivated tomato (Darwin, Knapp & Peralta, 2003; The Tomato Genome Consortium, 2012).

*S. pimpinellifolium* is native to areas of low elevation on the western slopes of the Andes in Peru and Equador (Grandillo et al., 2011). It is the proposed nearest wild relative to the cultivated tomato (The Tomato Genome Consortium, 2012) and is the only red-fruited wild species. *S. pimpinellifolium* has been used to introduce traits such as disease resistance and improved soluble solids into cultivated tomato (Grandillo et al., 2011). The other two wild species, *Solanum galapagense*, along with the closely-related *Solanum cheesmaniae*, are perennials endemic to the Galápagos Islands and comprise the only two orange-fruited tomato species. *S. galapagense* was only recently recognized as a separate species from *S. cheesmaniae*. It was previously classified as *S. lycopersicon cheesmaniae* L. Riley var. *minor* (Hook.f) (Darwin, Knapp & Peralta, 2003) and there is debate based on genetic variation between the species that questions whether they should be classified as morphotypes rather than separate species (Lucatti et al., 2013). *S. galapagense* and *S. cheesmaniae* have been used to a limited degree in breeding programs, mainly to improve salt tolerance and soluble solids (Grandillo et al., 2011). Orange fruit color in these two species is due to a dominant variant of the *B* gene that results in 5 to 10-fold increase in *β*-carotene in comparison to red fruit (Ronen et al., 2002). These species have other phenotypic differences from cultivated tomato including scent, pathogen response, trichomes, and leaf morphology (Darwin, 2009).

The relative phylogenetic positions of *S. lycopersicum, S. galapagense, S. cheesmaniae*, and *S. pimpinellifolium* are currently unresolved (Grandillo et al., 2011). Several different tree topologies have been inferred for the species in recent literature using various methods (Grandillo et al., 2011). In recently diverged species such as these, phylogenetic discordance can be prevalent (Ané, 2011) due to both incomplete lineage sorting of ancestral polymorphism and introgression from other species. Introgression is expected to make an especially strong contribution to phylogenetic discordance in tomato species, due to the use of wild species in the development of various *S. lycopersicum* cultivars. In particular, the sequenced tomato *S. lycopersicum* ‘Heinz 1706’ (H1706) is known to have *S. pimpinellifolium* in its parentage (Ozminkowski, 2004; Labate & Robertson, 2012; The Tomato Genome Consortium, 2012). Interspecific hybridization also occurs in wild populations of tomato along hybrid zones (Nakazato & Housworth, 2010) and also as evidenced by *S. lycopersicum* var *cerasiforme*, which is purportedly the result of crossing between *S. lycopersicum* and *S. pimpinellifolium* (Ranc et al., 2008). In contrast, *S. galapagense* and *S. cheesmaniae* have likely evolved in relative isolation, although *S. pimpinellifolium* and *S. lycopersicum* have been introduced to the Galápagos Islands in the past few decades (Darwin, 2009). Heirloom lines, which have existed prior to the implementation of major breeding programs, have been perpetuated mainly from lines of *S. lycopersicum* often by home gardeners, decreasing their likelihood of containing introgressions from wild species.

For this study, we have sequenced *S. galapagense* and the heirloom line *Solanum lycopersicum* ‘Yellow Pear’ (YP-1) (Goldman, 2008). Given the close relationship between *S. galapagense* and *S. cheesmaniae, S. galapagense* was chosen as a representative sample for the current study. These data were used in conjunction with coding sequence data from *S. pimpinellifolium* (The Tomato Genome Consortium, 2012), *S. corneliomuelleri* (Park et al., 2012), and *S. tuberosum* (Potato Genome Sequencing Consortium, 2011). Positively selected genes along the *S. lycopersicum* lineage were of interest since they may relate to domestication phenotypes. Using whole genome sequence from H1706, YP-1, *S. galapagense*, *S. pimpinellifolium, S. tuberosum*, and the H1706 reference genome sequence (The Tomato Genome Consortium, 2012), regions of divergence from cultivated tomato including structural variation were identified and the placement of *S. galapagense* on the *Solanum* phylogenetic tree was resolved for these genotypes. Also, a survey of genomic discordance was performed to gain a greater understanding of phylogenetic incongruence in newly diverged plant species. *S. lycopersicum* YP-1, a heirloom line that predates major tomato breeding programs was included as a negative control for introgressions from wild tomato species. All data and results from this study are available at the Sol Genomics Network site (http://solgenomics.net/) (Bombarely et al., 2011).

## Results

### Assembly statistics

Quality filtering and trimming of the paired-end reads yielded 462.7 million *S. lycopersicum* H1706 reads, 420.3 million YP-1 reads, 363.9 million *S. galapagense* reads, and 281.5 million *S. pimpinellifolium* reads (Table S1). Approximately 92.1% of the *S. lycopersicum* H1706 reads, 93.5% of the YP-1 reads, 89% of the *S. galapagense*, 88% of the *S. pimpinellifolium* reads mapped to the *S. lycopersicum* version 2.40 genome assembly giving 39x, 45x, 32x, and 25x coverage and covering 99.2%, 99.3%, 95.4%, and 95% of the tomato genome respectively, after mapping quality filtering and duplicate read removal (Table S1). Gaps were calculated as regions without read coverage that were not gaps in the *S. lycopersicum* H1706 scaffolds. Gap total size ranged from 5.4 Mbp for YP-1 to 38.9 Mbp for *S. pimpinellifolium* (Table S1).

In addition, *de novo* assemblies were produced for each non-reference genome (Table S2). By comparing assemblies generated from a range of k-mer values, the best k-mer values were found to be 63, 57, and 51, for YP-1, *S. galapagense*, and *S. pimpinellifolium* respectively. Contigs greater than 200 bp were used for further analysis. The YP-1 assembly produced the largest contigs with an N50 of 25.2 kb totaling 716.7 Mbp of sequence while *S. pimpinellifolium* had the shortest with an N50 of 5 kb totaling 669.3 Mbp of sequence (Table S2).

### SNP and indel detection and effect on the genome

**Figure 1:**
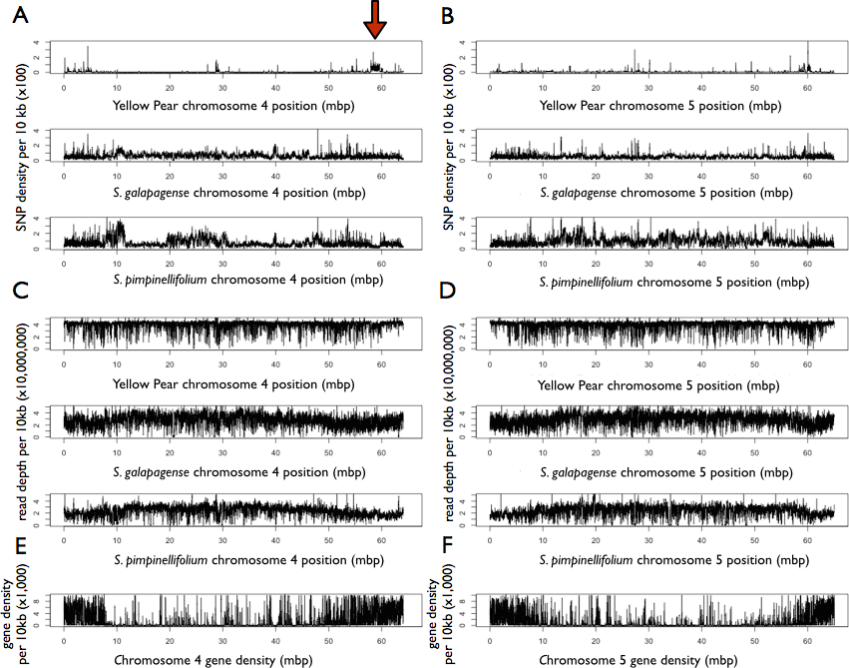
Feature density of Yellow Pear, *S. galapagense*, and *S.pimpinellifolium* in comparison to H1706. Red arrow points to putative introgression. (A) SNP density on chromosome 4 of sequenced genotypes. (B) SNP density on chromosome 5 of sequenced genotypes. (C) Read depth on chromosome 4 of sequenced genotypes. (D) Read depth on chromosome 5 of sequenced genotypes (E) Gene density on chromosome 4 based on *H1706* annotations (F) Gene density on chromosome 5 based on H1706 annotations.

Over 500,000 single nucleotide polymorphisms (SNPs) were found between YP-1 and H1706 (Table S3). *S. galapagense* was found to have approximately 4.7 million SNPs, whereas *S. pimpinellifolium* had 6 million when compared to H1706 (Table S3). Variation in SNP density was found across the genome, and was found to differ between chromosomes and genotypes (Fig. 1 and Fig. S1). In particular, regions on chromosomes 4 (∼59 Mbp) and 11 (∼4 Mbp) show reduced SNP density in *S. pimpinellifolium* and elevated density in YP-1 (Fig. 1 and Fig. S1). A large assembly coverage gap in *S. pimpinellifolium* located at approximately 11 Mbp on chromosome 1 is found at the position of the tomato self-incompatibility locus (Tanksley & Loaiza-Figueroa, 1985) (Fig. S1). Large assembly coverage gaps were also detected in *S. pimpinellifolium* on chromosomes 3 (∼37 Mbp), 8 (∼40 Mbp), 10 (∼30 Mbp), and *S. galapagense* chromosomes 8 (∼16 Mbp), and 12 (∼60 Mbp) (Fig. S1). As expected, more SNPs were found in noncoding regions than coding regions (Table S3). SNPs were found in approximately 0.05%, 0.5%, and 0.8% of the YP-1, *S. galapagense*, and *S. pimpinellifolium* intergenic regions respectively, while affecting only 0.04%, 0.3%, and 0.4% of the coding regions of these genomes (Table S3). A total of 3,418 YP-1, 20,447 *S. galapagense*, and 12,143 *S. pimpinellifolium* genes were found to have nonsynonymous SNPs associated with them. Additionally, 242,165 SNPs were identified using the aligned Illumina data from H1706 to the reference H1706 v 2.40 assembly, of which 225,625 were predicted to be heterozygous with the reference genome (please see solgenomics.net for vcf file).

Approximately 50,000 indels were found between YP-1 and H1706, 350,000 between *S. galapagense* and H1706, and 520,000 between *S. pimpinellifolium* and *S. lycopersicum* H1706 (Table S4). Indels were more prevalent in noncoding regions (Table S4). Indels in coding sequence were found in a total of 595 YP-1 genes, 3,493 *S. galapagense* genes, and 3,645 *S. pimpinellifolium* genes. Additionally, 41,776 indels were identified between the H1706 sequence and H1706 v 2.40, 4,716 of which were heterozygous (please see solgenomics.net for vcf file).

### Structural variation

**Figure 2:**
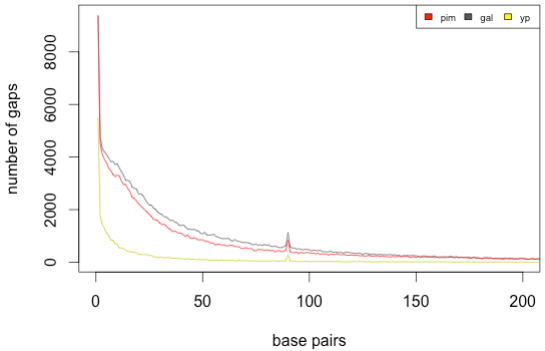
Putative deletion size distribution in combined assemblies.

To determine the nature of regions where reads from YP-1, *S. galapagense*, or *S. pimpinellifolium* could not map to the H1706 genome, but H1706 reads could map, these regions were further analyzed for each species. Regions lacking coverage in the H1706 mapping are mainly scaffolding gaps in the H1706 reference genome. Gap size distribution was similar between *S. galapagense* and *S. pimpinellifolium* with less gaps found in YP-1 (Fig. 2), with all genotypes having a peak at 90 bp. Since gaps could be missing regions or divergent regions where short reads cannot map, *de novo* contigs assembled from the wild and heirloom species reads were mapped to the reference genome to determine if they covered gap regions. Approximately 3.3% of YP-1, 3.7% of *S. galapagense*, and 6.0% of *S. pimpinellifolium* contigs did not map with greater than 90% id. A small number of these contigs contained many repeats or matched plastid, mitochondrial, or vector DNA (Table S5). After removal of gaps covered by *de novo* contigs, a total of 2.4 Mbp of YP-1, 13.8 Mbp of *S. galapagense*, and 21.6 Mbp of *S. pimpinellifolium* was putatively deleted relative to H1706. The largest gap in each species was 12.7 kbp on chromosome 12 for YP-1, 41 kb on chromosome 12 of *S. galapagense*, and 38.7 kbp on chromosome 10 of *S. pimpinellifolium* (File S1). Deleted genes were determined as genes that were at least 90% contained in putative gaps and had no matches in *de novo* contig assemblies. A total of 13 genes from YP-1, 87 genes in *S. galapagense*, and 157 in *S. pimpinellifolium* were found to have no coverage in either the small read mapping or contig mapping (Table S6). Many of these genes were classified as disease resistance-related proteins or lacked a predicted function (Table S6).

Two small insertions of 130 bp were predicted in *S. pimpinellifolium* in reference to H1706 on chromosomes 4 and 10, but these were not well supported (Table S7). No insertions larger than 20 bp could be predicted in the other genotypes relative to H1706 with these datasets.

### Patterns of gene evolution in *Solanum*

To determine the average nucleotide substitution rate amongst coding sequences, aligned sequence from 32,982 *S. galapagense* genes and 32,795 *S. pimpinellifolium* genes was used to generate estimates of nonsynonymous (dN) and synonymous (dS) substitution rates in reference to YP-1 (Table 1). H1706 was not considered in the analysis since introgression from wild species could bias the analysis. Missing genes or genes containing stop codons were removed from the analysis. *S. pimpinellifolium* had a larger average dS than *S. galapagense* (Table 1). The number of synonymous substitutions per synonymous site ranged from 0 to 0.5655 for *S. galapagense*, and 0 to 0.3403 for *S. pimpinellifolium.* Nonsynonymous substitutions per nonsynonymous site ranged from 0 to 0.2106 in *S. galapagense* and 0 to 0.1105 in *S. pimpinellifolium*.

**Table 1:** Pairwise estimates of nonsynonymous (dN), synonymous (dS) mean substitution rate. Calculations are in comparison with *S. lycopersicum* ‘Yellow Pear’ and are based on 8,578 orthologous coding sequences for numbers not in parenthesis. Numbers in parenthesis are based on all usable coding sequences.

Species	dN	dS	*ω* ^a^
*S. galapagense*	0.0012 ± 0.0019	0.0037 ± 0.0059	0.3535 ± 2.0205
	*(0.0029 ± 0.0058)*	*(0.0052 ± 0.0117)*	*(0.5191 ± 3.2039)*
*S. pimpinellifolium*	0.0013 ± 0.0022	0.0043 ± 0.0065	0.4305 ± 2.9802
	*(0.0033 ± 0.0062)*	*(0.0064 ± 0.0126)*	*(0.5300 ± 3.3742)*
*S. corneliomuelleri*	0.0037 ± 0.0041	0.0151 ± 0.0123	0.3219 ± 1.0219
*S. tuberosum*	0.0332 ± 0.4361	0.1306 ± 1.3060	0.2386 ± 0.3127

**Notes.**

a Maximum likelihood estimate, values >99 removed.

**Figure 3:**
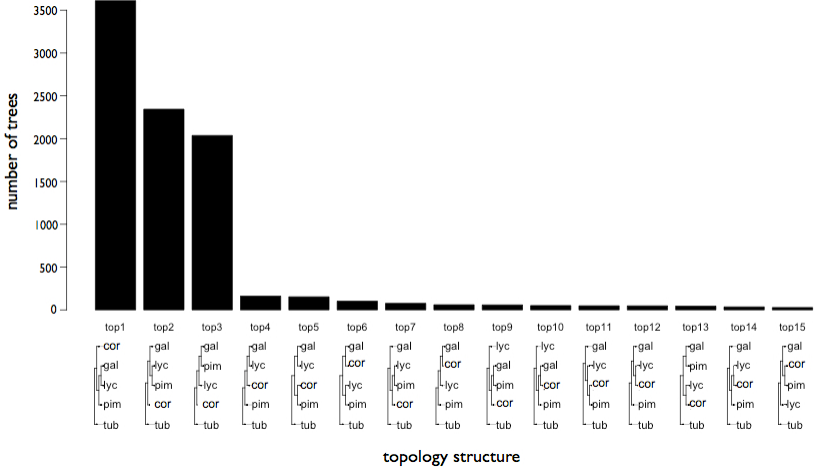
Gene trees inferred from coding sequence of 8,796 Solanum species genes. Phylogenetic trees were derived using maximum likelihood and were supported in at least 75 of 100 bootstrap replicates.

The coding sequence from 8,857 orthologous genes that could be aligned with confidence between YP-1, *S. galapagense, S. pimpinellifolium, S. corneliomuelleri*, and *S. tuberosum* were analyzed to infer gene tree topology using maximum likelihood. The majority of trees (3,611) supported tree topology 1 which groups *S. lycopersicum* and *S. galapagense*, suggesting these two species may be more closely related, although two other tree topologies were also well supported, albeit to a lesser degree (2,344 and 2,037 trees) (Fig. 3). The genes were then subjected to site-branch selection tests along the *S. lycopersicum* lineage. Stop codons were found in at least one of the species for 288 genes and these were removed from further analysis.

A total of 25 genes showed evidence of a faster rate of evolution along the *S. lycopersicum* lineage (File S2 and Table S8). Many of these genes have predicted function in adaptive or domestication phenotypes such as pathogen and abiotic stress response, cell division, and carbohydrate metabolism (Table S8).

Species divergence time estimates calculated based on dS values from 3,611 genes fitting topology 1 suggest a divergence estimate for *S. lycopersicum* and *S. pimpinellifolium* of 0.44 MYA (Table 2). Using coalescence-based divergence estimates of 8 genes fitting topology 1, a similar divergence of 0.45 MYA was obtained (Table 2), although hybridizations signatures were apparent between the species (Fig. S2). A subsample of genes was used since these analyses are not easily scaleable to a large number of loci. The signatures support the hypothesis of recent hybridizations between the following groups: (1) *S. lycopersicum* and *S. galapagense*, (2) *S. lycopersicum* and *S. pimpinellifollium*, and (3) *S. galapagense* and *S. pimpinellifollium*. A more recent divergence of 0.19 MYA, using dS values, and 0.25, using the coalescence method, was estimated for *S. lycopersicum* and *S. galapagense* (Table 2).

**Table 2:** Divergence time estimates of selected species. Calculations for Global Clock Method based on pairwise silent site substitutions for 3,611 genes. Calculations for coalescence method were performed with 8 genes. All genes used in calculations fit gene tree topology 1. Divergence date estimates are in reference to H1706.

Species	dS	Divergence Date (MYA)^a^	**Divergence Date (MYA)** ^b^
*S. galapagense*	0.0024 ± 0.0038	0.19	0.25
*S. pimpinellifolium*	0.0053 ± 0.0066	0.44	0.45
*S. corneliomuelleri*	0.0166 ± 0.0126	1.38	1.54
*S. tuberosum*	0.1335 ± 1.2383	11.07	NA

**Notes.**

a Based on global clock method.

b Based on coalescence method.

MYA million years ago

### Genomic phylogenetic discordance

To look at genome-wide phylogenetic discordance, whole genome alignments were created with H1706, YP-1, *S. galapagense, S. pimpinellifolium*, and *S. tuberosum*. A total of 781.5 Mbp of the H1706 genome was represented in the alignment. The alignments were then partitioned into 100 kb windows resulting in 8,275 loci, since some alignments were shorter than 100 kb.

**Figure 4:**
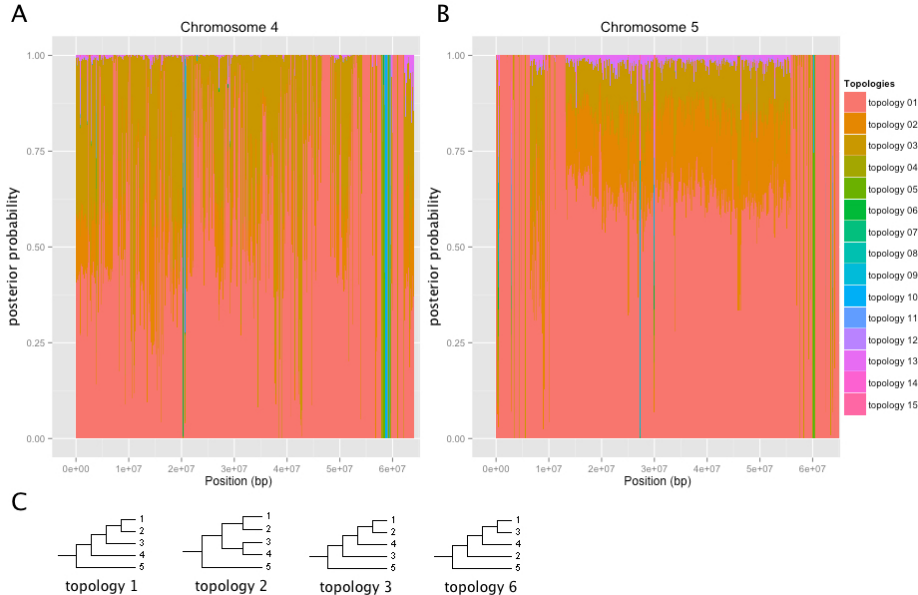
Tree topologies across selected chromosomes of H1706. Coordinates are based on the H1706 reference genome. Posterior probabilities are shown for each tree. (A) Chromosome 4. (B) Chromosome 5. (C) Predominant tree topologies. 1 = YP; 2 = H1706; 3 = *S. galapagense*; 4 = *S. pimpinellifolium*; 5 = *S. tuberosum.*

Trees for each genome partition were constructed using Bayesian phylogenetic analysis. A total of 217 loci contained gaps in the alignment and the topology could not be deduced. A total of 2,227 loci covering 27% of the H1706 genome supported topology 1 with a posterior probability of 0.9 or greater (Fig. 4, File S3, and Fig. S3) grouping *S. galapagense* closer to *S. lycopersicum* than to *S. pimpinellifolium* (Fig. 4, File S3, and Fig. S3). Topology 3, which clusters the two *S. lycopersicum* varieties more closely to *S. pimpinellifolium*, was found with a posterior probability of 0.9 or greater at 224 loci covering 2.8% of the H1706 genome (Fig. 4. File S3, and Fig. S3). Overall, the predominant tree topology was topology 1 which was the best supported topology at 72.9% of the genome. Topology 3 was the second most prevalent tree and supported at 19.7% while topology 2 was found at 5.6% of the genome.

A total of 82 loci constituting 0.9% of the H1706 genome best supported topologies indicative of introgression in H1706, placing *S. pimpinellifolium* closer to H1706 than YP-1. This includes an introgression of 19.9 kb starting at the beginning of chromosome 9 which is linked to *Ve1* (Solyc09g005090) and *Ve2* (Solyc09g005080), involved in Verticillium wilt resistance (Kawchuk et al., 2001) and a 1.1 Mbp region on chromosome 11 that contains the *I* gene, which confers resistance to Fusarium wilt (Scott, Agrama & Jones, 2004) (Table S9). An additional 1.7 Mbp region on chromosome 4 containing 172 gene models was found to be introgressed in H1706, although, to date, no known disease resistance genes are found here (Fig. 4 and Table S9).

By using functional predictions for the gene models predicted within the chromosome 11 introgression, four TIR-NB-LRR resistance proteins were identified (Table S9). Since *I2* is known to be a protein of this type, these genes are likely candidates for *I* (Scott, Agrama & Jones, 2004). One of the candidates, Solyc11g011080, was found to have a frameshift mutation and possible splice site mutation in YP-1, while retaining the H1706 reading frame in *S. pimpinellifolium*.

## Discussion

Here, we present two new genome assemblies: the wild tomato species, *S. galapagense*, and an heirloom variety, *S. lycopersicum* ‘Yellow Pear.’ We determined variation by comparing these two assemblies, as well as the published assemblies of *S. pimpinellifolium* and the reference H1706 genome. While a difference in SNP count was found between this study and a previous study for *S. pimpinellifolium* (The Tomato Genome Consortium, 2012), the same SNP calling pipeline was used for all genotypes in this study, so estimates of variations across species should not be biased. The homozygous SNPs and indels, which were identified by mapping reads from H1706 to the reference genome from this genotype, are likely errors in the reference sequence. Heterozygous sites in H1706 were identified. It is also possible that 454 sequencing used in the reference assembly introduced indel errors. Slightly more gaps were found in the H1706 assembly than in the YP-1 assembly, which could be related to newer technologies used for library preparation for the sequencing of YP-1. The gap peak found at 90 bp is due to scaffolding gaps in the H1706 reference assembling. Putative divergent regions in the assemblies are likely not repetitive regions or other regions where reads map poorly to the reference genome, since these regions would have been removed from further analysis based on gaps in the H1706 reference-guided assembly. Large insertions could not be predicted with accuracy, likely due to the use of only short insert size paired-end libraries. Based on the total length of the *de novo* assemblies and divergent regions, it is likely that the genome size of *S. galapagense* is comparable to *S. lycopersicum*, although earlier studies suggested a possibly smaller genome (Arumuganathan & Earle, 1991). *S. pimpinellifolium* may have a smaller genome than H1706, based on gap sizes and kmer assessment.

Omega values are a method of determining selection at a coding locus. These values tend to decrease with evolutionary distance (Wolf et al., 2009) and a similar result was obtained based on the coding sequence analysis of *S. pimpinellifolium*, *S. corneliomuelleri*, and *S. tuberosum*. Since the omega value is a ratio derived from scaling dN by dS, omega values can be artificially inflated if synonymous mutations are not neutral and also as a factor of short branch length. The latter is a likely explanation for our results, as species in the tomato clade have a very recent divergence. For example, one of the more divergent wild tomato species, *S. pennellii*, has an estimated divergence from *S. lycopersicum* of only 7 MYA (Nesbitt & Tanksley, 2002). Our results suggest a more recent divergence of tomato from its closest wild relatives, giving further evidence of short branch length. Our results are similar to a previous estimate of ∼1 MYA for the divergence of *S. lycopersicum, S. cheemaniae*, and *S. pimpinellifolium*, which was based on a smaller gene sample size (Nesbitt & Tanksley, 2002). Interestingly, a much larger number of *S. galapagense* genes are affected by nonsynonymous substitutions, which could be due to the fixation of slightly deleterious alleles due to drift acting strongly on a small population size during initial colonization of the Galápagos Islands. However, other mutations such as stop codon, indels, and other deleterious mutations are not inflated, suggesting this increase in nonsynonymous substitutions could be do to positive selection. Nonsynonymous substitutions per nonsynonymous site has a larger range in *S. galapagense* also likely due to drift or selection.

Only 25 genes were detected in this study that are candidates for selection along the *S. lycopersicum* lineage. Many factors relevant to this data set likely play a role in these results. The reduced gene set only includes genes with putative orthologs that fit strict criteria and contained matches in all species studied. In particular, the *S. corneliomuelleri* transcriptome dataset contained only 50% of the total number of expected genes based on tomato annotations. As a result, this data set is likely a biased sample including predominantly genes that are more conserved across Solanum species. Indeed, a study analyzing a larger sample of 11,221 genes, found a total of 51 genes to be positively selected (Koenig et al., 2013). Moreover, the short branch length of members of the tomato clade impedes detection of differential rates of evolution. An average dS of 0.05 is necessary for detection of lineage-specific selection, meaning there is little statistical power to detect selection along a lineage in this group (Yang, 2007). To detect selection within the tomato clade, alternative selection detection methods, such as McDonald–Kreitman tests (McDonald & Kreitman, 1991) involving intraspecific data, may prove useful, as well as a larger sample size of genes.

Whole genome phylogenies proved useful to detect topological discordance in these recently diverged plant species. Since a greater number of SNPs occur in non-coding genomic regions, higher phylogenetic signal may be achieved with genomic alignments, rather than only coding sequence. In our study, most regions of the H1706 genome where phylogenies do not fit the majority rules species tree did fit a pattern expected from incomplete lineage sorting; for example, grouping both *S. lycopersicum* varieties closer to *S. pimpinellifolium*. Incomplete lineage sorting is also supported by the nearly equal frequency of two secondary trees in the coding sequence phylogenies (Patterson et al., 2012). These results are expected when speciation has occurred in a short period of time from an ancestral population with greater diversity, which is likely the case in this study. A secondary cause of phylogenetic discordance was found in regions of introgression from *S. pimpinellifolium* in the H1706 genome and could be ascertained by the inclusion of the heirloom YP-1 as a control. Genome-wide phylogenies, as well as SNP density patterns on chromosomes 4, 9, and 11, support introgression of a *S. pimpinellifolium* in the H1706 genome. Some smaller putative introgressions were found on chromosomes 1, 2, 3, 5, 6, 8, and 10. Additionally, an overlapping region on chromosome 4 was found in comparisons to an inbred line to H1706 further supporting an introgression in H1706 at this location (Menda et al., 2014). These regions are in concordance with previous introgression predictions (The Tomato Genome Consortium, 2012) and the known H1706 pedigree (Ozminkowski, 2004). There are also several regions of high SNP density across the chromosomes that do not correspond to regions identified as introgressions in the tree topologies, suggesting these are regions of high variability. For example, *S. pimpinellifolium* chromosome 1 has a region of high SNP density on either side of the self-incompatibility locus. Self-incompatibility loci are known to exhibit high polymorphism and rearrangement (Wang et al., 2003) and this is evidenced by the lack of read coverage in the immediate area between *S. pimpinellifolium* and H1706. This result would also suggest the sequenced *S. pimpinellifolium* has a self-incompatibility haplotype that is different from H1706, YP-1, and *S. galapagense*. Also, it is important to keep in mind, while H1706 is known to have *S. pimpinellifolium* in its parentage, the specific genotype analyzed here may be different than the H1706 parental genotype, resulting in variation between the introgressed segments in H1706.

Despite extensive phylogenetic discordance, by using coding sequence and whole genome sequence data, we were able to ascertain a predominant species tree for the varieties in this study. *S. galapagense* is more closely related to *S. lycopersicum* than *S. pimpinellifolium* as supported in some previous studies, one of which includes a different *S. galapagense* genotype than what was used in our study (Grandillo et al., 2011; Koenig et al., 2013). It is possible by sampling the spectrum of variation in *S. pimpinellifolium* a more closely related genotype to cultivated tomato may be found. Sequencing of additional genotypes of these species will help shed light on the evolution of domesticated tomato.

## Conclusions

H1706 provides an excellent reference for genome assembly of its nearest wild relatives and allows for efficient genome analysis. Using this reference genome, we have determined areas of variation across closely related tomato species and found candidate genes that could be involved in domestication, crop improvement, or adaptation to new environments. Genome-wide phylogenies support this *S. galapagense* genotype as the closest wild relative of cultivated tomato in our study. The sequenced tomato is expected to have wild introgressions and we have successfully delimited candidate introgression regions from wild species. This method may also be useful in detecting candidate regions for breeding purposes as well as conservation biology, since wild species may be threatened due to introgression from cultivated tomatoes (Darwin, Knapp & Peralta, 2003; Darwin, 2009).

## Methods

### Solanum lines and libraries

*S. galapagense* genotype LA0436 was obtained from the Tomato Genetic Resource Center (TGRC; http://tgrc.ucdavis.edu/) and *S. lycopersicum* ‘Yellow Pear’ (YP-1) was obtained from the Martin Lab. Genomic DNA was prepared using a modified version of a protocol described previously (Zhang et al., 1995) using precipitation and CsCl purification instead of agarose bead imbedding. Samples for *S. galapagense* were sent to the Life Science Core Laboratory Center at Cornell University (Ithaca, NY) for library preparation and sequencing. YP-1 was sent to Genomics Resources Core facility at Weill Cornell Medical College (New York, NY) for library preparation and sequencing. *S. pimpinellifolium* genotype LA1589 and *S. corneliomuelleri* genotype LA0103 were sequenced by the Lippman Lab at Cold Spring Harbor (Park et al., 2012; The Tomato Genome Consortium, 2012). H1706 and *Solanum tuberosum* sequence is publicly available (Potato Genome Sequencing Consortium, 2011; The Tomato Genome Consortium, 2012). H1706 Illumina paired-end data from libraries 090617, 090619, and 090701_SNPSTER5B was provided by Syngenta.

### lllumina sequencing

Sheared genomic DNA from *S. galapagense* was run on 2 lanes of an Illumina HiSeq 2000 (Illumina, San Diego, California, USA). Read length was 100 base pairs (bp) and insert size was 200 bp. In addition, sheared genomic DNA was run on 7 lanes of an Illumina GA II using the mate pair module. Genomic DNA from YP-1 was run on 1 lane of an Illumina HiSeq 2000 and the resulting sequence was 100 bp in length with an insert size of 300 base pairs. *S. galapagense* and YP-1 sequence was submitted to the NCBI Small Read Archive (SRA) as experiment numbers SRX520161 and SRX521582. Data and output from this study can be accessed through Solgenomics at ftp://ftp.solgenomics.net/genomes/.

### Sequence assembly

Reads were inspected for quality using FastQC (http://www.bioinformatics.babraham.ac.uk/projects/fastqc/) and rechecked after cleaning. Cleaning was performed with fastq-mcf (https://code.google.com/p/ea-utils/wiki/FastqMcf). Reads were mapped to the H1706 reference assembly v 2.40 using a tiered approach with an initial round of BWA (Li & Durbin, 2009) mapping followed by Novoalign (http://novocraft.com/) for the remaining discordant and unmapped reads. Duplicate reads and reads with a mapping quality less than 30 were removed for variation analysis with Picard (http://picard.sourceforge.net) and Samtools (Li et al., 2009), respectively. A mapping quality of 30 means for approximately every 1000 mapped reads, one will be mapped incorrectly.

Whole genome *de novo* assemblies of *S. galapagense*, *S. pimpinellifolium*, and YP-1  were created using SOAP *de novo* version 1.05 (Li et al., 2008). Assemblies were produced using a kmer range between 25 and 63. Scripts supplied with the SOAP *de novo* package were used for error correction and gap filling of the scaffolds. Reads that did not map or did not pair properly in the reference-guided assembly were mapped to the *de novo* assembled contigs for each genome. Contigs that had an above average number of reads mapped to them were further analyzed (see next section).

### Variation discovery

SNPs and indels 15 base pairs and smaller were detected using the GATK recommended best practices (McKenna et al., 2010). Since a suitable dataset was not available for base quality calibration, one was generated by pooling high quality SNPs from both *S. galapagense* and *S. pimpinellifolium*. Snpeff was used to determine the effect of each SNP and indel in the genome and determine zygosity (Cingolani et al., 2012). Putative deleted regions were detected by finding regions that had no sequence coverage and did not overlap with gaps in the reference assembly using Bedtools (Quinlan & Hall, 2010). Only gaps greater than 15 base pairs and not found on chromosome 0 were used for further analysis. These regions were compared to the mapping assembly of H1706 and matching gaps were removed from further analysis. BLAT (Kent, 2002) with default values (sequence identity 90%) was used to map *de novo* assembled contigs greater than 200 bp from each genotype to the reference genome. The best hit was determined by using scripts included with the BLAT package. Unmapped contigs were processed by Seqclean (https://sourceforge.net/projects/seqclean/) to identify matches to *S. lycopersicum* plastid or mitochondrial DNA (The Tomato Genome Consortium, 2012), plant pathogen sequence found in Comprehensive Phytopathogen Genomics Resource (CPGR) (Hamilton et al., 2011), vector sequence found in UniVec database (http://www.ncbi.nlm.nih.gov/VecScreen/UniVec.html), or contigs that were low complexity. Putative deletions were confirmed if *de novo* assembled contigs did not map to regions not covered in the reference-guided assemblies. Bedtools (Quinlan & Hall, 2010) was used to identify genes found at least 90% in deleted regions. BLAT (Kent, 2002) was used to search for orthologs of these genes in the *de novo* assemblies. Genes with hits covering less than 50% of the gene and not the top match in reciprocal BLAT (Kent, 2002) output were considered deleted. Breakdancer v1.1 (Chen et al., 2009) was used to predict insertions greater than 15 base pairs for insertion analysis.

### Coding sequence analysis

Predicted coding sequence from *S. galapagense*, and *S. pimpinellifolium* was used for pairwise comparisons to YP-1. Only genes with no stop codons predicted within the gene sequence were used. Coding sequence was predicted using H1706 annotation version ITAG2.3 (The Tomato Genome Consortium, 2012). Coding regions were first reverse translated and aligned using ClustalW (Thompson, Gibson & Higgins, 2002). Alignments containing premature stop codons were discarded. Pairwise maximum likelihood comparisons were performed to determine nonsynonymous and synonymous substitution rates using the codeml package of PAML version 4.5 (Yang, 2007).

Predicted coding sequence of genes from YP-1, *S. galapagense*, *S. pimpinellifolium*, *S. corneliomuelleri*, and *S. tuberosum* were subjected to phylogenetic analysis. Coding sequence with at least 50% *S. lycopersicum* gene coverage was selected as input. BLAST (Altschul et al., 1990) was used to find putative *S. lycopersicum* orthologs in *S. tuberosum* coding sequence. These matches were then used as a query for a reciprocal BLAST (Altschul et al., 1990) to the *S. lycopersicum* genome. Any hits that were not one-to-one matches were discarded. Alignments were calculated as above. The underlying phylogeny was calculated for each gene using DNAml with the Kimura model and 100 bootstrap replicates using PhygOmics (Bombarely et al., 2012). Pairwise estimates of *ω* were calculated using the codeml package of PAML (Yang, 2007). Codeml (Yang, 2007) was also used to perform a branch-site test to detect positive selection along the *S. lycopersicum* lineage. The maximum likelihood value from the alternative model allowing sites to evolve under positive selection was compared to the value from the null model in which no selection occurs. The null model was rejected if 2 times the difference between the log likelihood values was larger than 2.71 at the 5% significance level.

Divergence dating was estimated by assuming a nuclear gene substitution rate of 6.03 × 10-9 dS per site per year and dividing dS by 2 times the substitution rate (Nesbitt & Tanksley, 2002). These estimates were compared to coalescent-based estimates using *BEAST (Drummond & Rambaut, 2007). Only genes fitting the predominant gene tree topology were used in the calculations. Six gene clusters were used for this analysis (homologous genes to the reference gene models: Solyc02g081560, Solyc02g093130, Solyc04g054810, Solyc04g078200, Solyc06g009630, Solyc09g013140). Mega 6.06 (Tamura et al., 2013) was used to determine the best model for 6 of the genes as Jukes and Cantor based on BIC score for each gene. Monte Carlo Markov Chains (MCMC) of 100,000,000 generations were used to perform this analysis. See File S4 for Beast configuration parameters. DensiTree (Bouckaert, 2010) was used to visualize tree set output.

### Whole genome phylogeny

Genomes for YP-1, *S. galapagense*, and *S. pimpinellifolium* were created by substituting SNPs and masking gaps in coverage into the reference assembly. Repeat masking was performed using RepeatMasker (Smit, Hubley & Green, 1996–2010) and a tomato-specific repeat dataset (The Tomato Genome Consortium, 2012). Whole genome multiple sequence alignment were generated for H1706, YP-1, *S. galapagense*, *S. pimpinellifolium*, and *S. tuberosum* using Mercator and Mavid (Dewey, 2008). Each of the 8,275 loci was analyzed using MrBayes (Ronquist & Huelsenbeck, 2003). The analysis of each locus used 3 independent runs, each having 1 cold and 2 hot chains with a temperature spacing of 0.25, a run length of 200,000 generations, a burn-in fraction of 0.2, and a sampling frequency of 100. The Gelman–Rubin psrf convergence diagnostic (Gelman & Rubin, 1992) based on log-likelihoods was calculated for each locus analysis, with the result that psrf <1.05, indicating good convergence, for 99.9% of the loci. Trees were rooted using *S. tuberosum* as an outgroup. Tree locations were mapped to H1706 genomic coordinates.

## Supplemental Information

10.7717/peerj.793/supp-1 Table S1 Illumina read alignment to H1706 reference assembly and genome coverage metrics *S. gal*, *S. galapagense*; *S. pim*, *S. pimpinellifolium.*

10.7717/peerj.793/supp-2 Table S2 *De novo* assembly metrics Illumina reads were assembled using SOAP *de novo*. *S. gal*, *S. galapagense*; *S. pim*, *S. pimpinellifolium.*

10.7717/peerj.793/supp-3 Table S3 SNP positions in YP-1, *S. galapagense*, and *S. pimpinellifolium* Annotations are based on ITAG2.3 predictions for H1706. Numbers in parenthesis indicate percentage of the region containing SNP sites. *S. gal*, *S. galapagense*; *S. pim*, *S. pimpinellifolium*; nonsyn, nonsynonymous.

10.7717/peerj.793/supp-4 Table S4 Indel location in YP-1, *S. galapagense*, and *S. pimpinellifolium* reference-guided assemblies to H1706 Numbers in parenthesis indicate percentage of region containing indels. *S. gal*, *S. galapagense*; *S. pim*, *S. pimpinellifolium.*

10.7717/peerj.793/supp-5 Table S5 Structural variation in YP-1, *S. galapagense*, and *S. pimpinellifolium* assemblies in relation to H1706 Genes in divergent regions had a match to a *de novo* contig. Putative deleted genes had no matches to *de novo* contigs. *S. gal*, *S. galapagense*; *S. pim*, *S. pimpinellifolium.*

10.7717/peerj.793/supp-6 Table S6 Genes found to be missing in YP-1, *S. galapagense*, and *S. pimpinellifolium* assemblies

10.7717/peerj.793/supp-7 Table S7 Insertions found in *S. pimpinellifollium* assembly in reference to H1706

10.7717/peerj.793/supp-8 Table S8 PAML site-branch test analysis results for significant genes

10.7717/peerj.793/supp-9 Table S9 Genes predicted to be introgressed in H1706 genome

10.7717/peerj.793/supp-10 Figure S1 SNP Density and Read Coverage Coordinates based on H1706 assembly.

10.7717/peerj.793/supp-11 Figure S2 Beast results showing consensus trees Uncertainty of tree topology is displayed by finer lines within the best supported tree.

10.7717/peerj.793/supp-12 Figure S3 Whole genome phylogeny results for all chromosomes

10.7717/peerj.793/supp-13 File S1 Gaps in reference-guided assemblies Files are in BED format. *gal*, *S. galapagense*; *pim*, *S. pimpinellifolium*, yp. YP-1

10.7717/peerj.793/supp-14 File S2 Fasta coding sequence alignments for genes found to be significant in PAML analysis

10.7717/peerj.793/supp-15 File S3 Posterior probabilities of tree topologies for each chromosomal position

10.7717/peerj.793/supp-16 File S4 Beast results

10.7717/peerj.793/supp-17 File S5 Whole genome phylogeny results for all chromosomes

10.7717/peerj.793/supp-18 File S6 Genes predicted to be in introgressions in H1706 genome

## Abbreviations

YP-1: Yellow Pear
H1706: Heinz 1706
Mbp: megabase pairs
SNPs: single nucleotide polymorphisms
dN: nonsynonymous substitutions per nonsynonymous site
dS: synonymous substitutions per synonymous site
MDL: minimum description length
TGRC: Tomato Genetic Resource Center
bp: base pairs
SRA: Small Read Archive
CPGR: Comprehensive Phytopathogen Genomics Resource
MCMC: Monte Carlo Markov Chains.

## Additional Information and Declarations

### DNA Deposition

The following information was supplied regarding the deposition of DNA sequences: GenBank SRA SRX520161 and SRX521582 Solgenomics: ftp://ftp.solgenomics.net/genomes/.
