# Supplementary material for: Comparative genomics and phylogenetic discordance of cultivated tomato and close wild relatives
Source: PeerJ. 2015 Feb 26;3:e793. doi: 10.7717/peerj.793 (PMC4358695; doi:10.7717/peerj.793)
Supplement: Table S1 — S. gal, S. galapagense; S. pim, S. pimpinellifolium. [file peerj-03-793-s001.docx]

**Supplemental Table S1 Illumina read alignment to H1706 reference assembly and genome coverage metrics.** *S. gal = S. galapagense*; *S. pim = S. pimpinellifolium.*

|  | ***S. lycopersicum*** | | **Wild species** | |
| --- | --- | --- | --- | --- |
| **Reference-guided assembly** | **H1706** | **YP-1** | ***S. gal*** | ***S. pim*** |
| filtered reads in millions | 462.7 | 420.3 | 363.9 | 281.5 |
| mapped reads *(%mapped)* | 426.1 *(92.1%)* | 393.1 *(93.5%)* | 324.7 *(89.0%)* | 247.7 *(88.0%)* |
| coverage depth^1^ | 39.3x | 45x | 32x | 25x |
| coverage of tomato gen | 99.2% | 99.3% ^2^ | 95.4% ^2^ | 95.0% ^2^ |
| no of gaps *(total size in mb)* | 76,276  *(5.9)* | 51,980  *(5.4)* ^2^ | 227,699 *(36.1)* ^2^ | 209,919 *(38.9)* ^2^ |
| no of gaps > 500 bp | 1,660 | 1,926 ^2^ | 10,751 ^2^ | 14,396 ^2^ |

^1^ after removal of duplicate reads and reads with mapping quality less than 30.

^2^ gaps in Heinz assembly (“N”) removed from calculation.
