# Supplementary material for: Comparative genomics and phylogenetic discordance of cultivated tomato and close wild relatives
Source: PeerJ. 2015 Feb 26;3:e793. doi: 10.7717/peerj.793 (PMC4358695; doi:10.7717/peerj.793)
Supplement: Table S2 — Illumina reads were assembled using SOAP de novo. S. gal, S. galapagense; S. pim, S. pimpinellifolium. [file peerj-03-793-s002.docx]

**Supplemental Table S2 *De novo* assembly metrics.** Illumina reads were assembled using SOAP *de novo*. *S. gal = S. galapagense*; *S. pim = S. pimpinellifolium.*

| **SOAP *de novo* assembly** | **YP-1** | ***S. gal*** | ***S. pim*** |
| --- | --- | --- | --- |
| Total length (bp) | 716,647,248 | 719,408,159 | 669,323,555 |
| Contig N50 (bp) | 25,157 | 5,969 | 5,005 |
| Average contig length (bp) | 4,897 | 1,785 | 1,784 |
| Number of contigs > 200 bp | 146,338 | 403,107 | 375,222 |
| Longest contig (bp) | 267,555 | 113,720 | 78,081 |
