# Supplementary material for: Comparative genomics and phylogenetic discordance of cultivated tomato and close wild relatives
Source: PeerJ. 2015 Feb 26;3:e793. doi: 10.7717/peerj.793 (PMC4358695; doi:10.7717/peerj.793)
Supplement: Table S3 — Annotations are based on ITAG2.3 predictions for H1706. Numbers in parenthesis indicate percentage of the region containing SNP sites. S. gal, S. galapagense; S. pim, S. pimpinellifolium; nonsyn, nonsynonymous. [file peerj-03-793-s003.docx]

**Supplemental Table S3 SNP positions in YP-1, *S. galapagense*, and *S. pimpinellifolium*.** Annotations are based on ITAG2.3 predictions for H1706. Numbers in parenthesis indicate percentage of the region containing SNP sites. *S. gal* = *S. galapagense*; *S. pim* = *S. pimpinellifolium*; nonsyn=nonsynonymous.

|  | **# of SNPs (% of region)*** | | |
| --- | --- | --- | --- |
| **SNP Location** | **YP-1** | ***S. gal*** | ***S. pim*** |
| Total SNPs | 539,406 *(0.07%)* | 4,665,765 *(0.6%)* | 6,016,177 *(0.8%)* |
| Intergenic | 339,858 *(0.05%)* | 4,318,821 *(0.5%)* | 5,589,364 *(0.8%)* |
| Genic | 42,117 *(0.04%)* | 346,944 *(0.3%)* | 426,813 *(0.4%)* |
| Genic - noncoding | 26,025 *(0.04%)* | 241,551 *(0.3%)* | 304,830 *(0.4%)* |
| Genic - coding | 16,092 *(0.04%)* | 105,393 *(0.3%)* | 121,983 *(0.3%)* |
| coding nonsyn. | 10,118 | 62,432 | 70,474 |
| coding synonymous | 15,457 | 40,622 | 40,703 |
| stop gained | 288 | 1,427 | 1,781 |
| stop lost | 144 | 497 | 541 |
| splice site donor | 49 | 271 | 320 |
| start lost | 64 | 258 | 326 |
| splice site acceptor | 55 | 251 | 255 |
| synonymous stop | 21 | 120 | 152 |
| nonsyn. start | 14 | 53 | 54 |

***** some SNPs are counted more than once since they are in more than one gene model
