# Supplementary material for: Comparative genomics and phylogenetic discordance of cultivated tomato and close wild relatives
Source: PeerJ. 2015 Feb 26;3:e793. doi: 10.7717/peerj.793 (PMC4358695; doi:10.7717/peerj.793)
Supplement: Table S4 — Numbers in parenthesis indicate percentage of region containing indels. S. gal, S. galapagense; S. pim, S. pimpinellifolium. [file peerj-03-793-s004.docx]

**Supplemental Table S4 Indel location in YP-1, *S. galapagense,* and *S. pimpinellifolium* reference-guided assemblies to H1706.** Numbers in parenthesis indicate percentage of region containing indels. *S. gal = S. galapagense*; *S. pim = S. pimpinellifolium.*

|  | **# of indels (% of region)** | | |
| --- | --- | --- | --- |
| **Indel Location** | **YP-1** | ***S. gal*** | ***S. pim*** |
| Total indels | 51,894 *(0.01%)* | 351,421 *(0.04%)* | 521,532 *(0.07%)* |
| Intergenic | 40,078 *(0.01%)* | 303,630 *(0.05%)* | 460,989 *(0.07%)* |
| Genic total | 11,816 *(0.01%)* | 47,791 *(0.04%)* | 60,543 *(0.06%)* |
| Genic - noncoding | 11,155 *(0.02%)* | 43,226 *(0.06%)* | 56,029 *(0.08%)* |
| Genic - coding | 661 *(0.0%)* | 4,434 *(0.01%)* | 4,514 *(0.01%)* |
| frame shift | 510 | 2,633 | 2,853 |
| codon insertion | 59 | 571 | 902 |
| codon deletion | 63 | 505 | 763 |
| change and insertion | 31 | 417 | 332 |
| change and deletion | 19 | 308 | 293 |
