# Supplementary material for: Comparative genomics and phylogenetic discordance of cultivated tomato and close wild relatives
Source: PeerJ. 2015 Feb 26;3:e793. doi: 10.7717/peerj.793 (PMC4358695; doi:10.7717/peerj.793)
Supplement: Table S5 — Genes in divergent regions had a match to a de novo contig. Putative deleted genes had no matches to de novo contigs. S. gal, S. galapagense; S. pim, S. pimpinellifolium. [file peerj-03-793-s005.docx]

**Supplemental Table S5 Structural variation in YP-1, *S. galapagense,* and *S. pimpinellifolium* assemblies in relation to H1706.** Genes in divergent regions had a match to a *de novo* contig. Putative deleted genes had no matches to *de novo* contigs. *S. gal = S. galapagense*; *S. pim = S. pimpinellifolium.*

|  | ***S. lycopersicum*** | **Wild Species** | |
| --- | --- | --- | --- |
|  | **YP-1** | ***S. gal*** | ***S. pim*** |
| unmapped contigs at 90% id | 11,841 | 15,067 | 22,346 |
| number matching plastid, mitochondrial, vector DNA, or low complexity | 188 | 32 | 1,226 |
| Uncovered regions (bp) | 951,969 | 13,812,215 | 21,599,274 |
| Genes in divergent regions | 3 | 18 | 16 |
| Putative deleted genes | 10 | 69 | 141 |
