# Supplementary figures and images for: Comparative genomics and phylogenetic discordance of cultivated tomato and close wild relatives
Source: PeerJ. 2015 Feb 26;3:e793. doi: 10.7717/peerj.793 (PMC4358695; doi:10.7717/peerj.793)

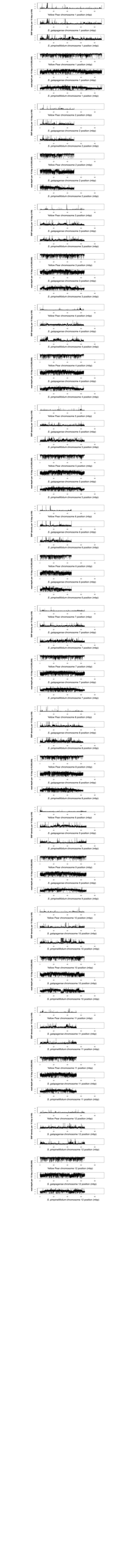

Supplement: Figure S1 — Coordinates based on H1706 assembly. [file peerj-03-793-s010.png]

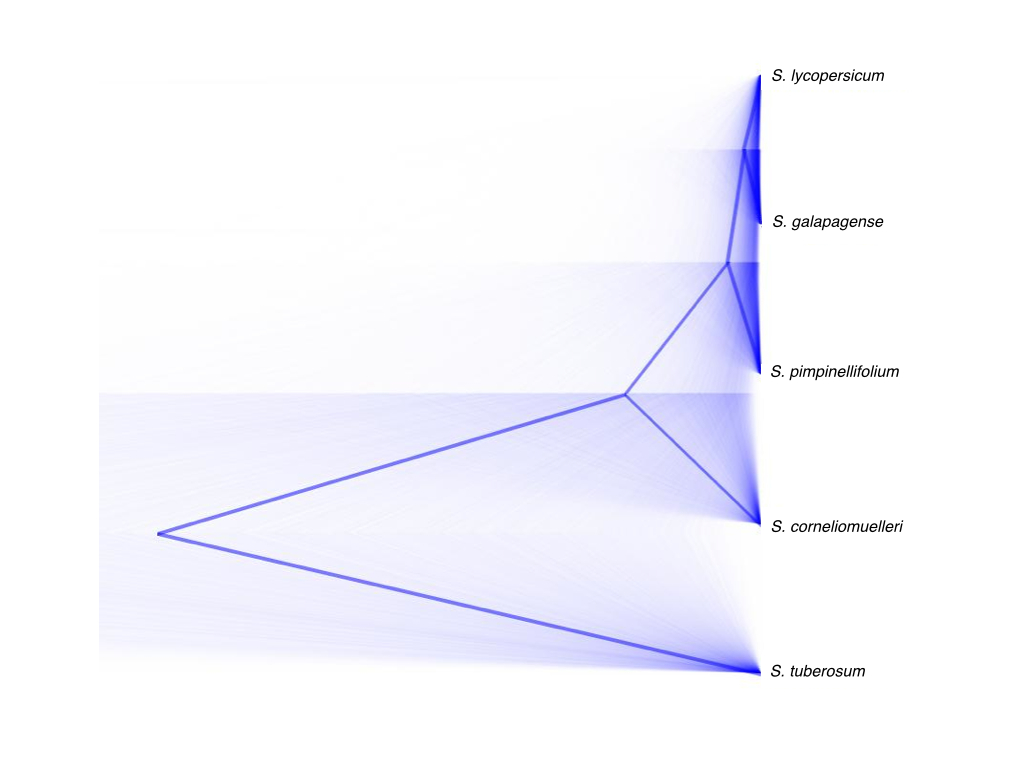

Supplement: Figure S2 — Uncertainty of tree topology is displayed by finer lines within the best supported tree. [file peerj-03-793-s011.png]

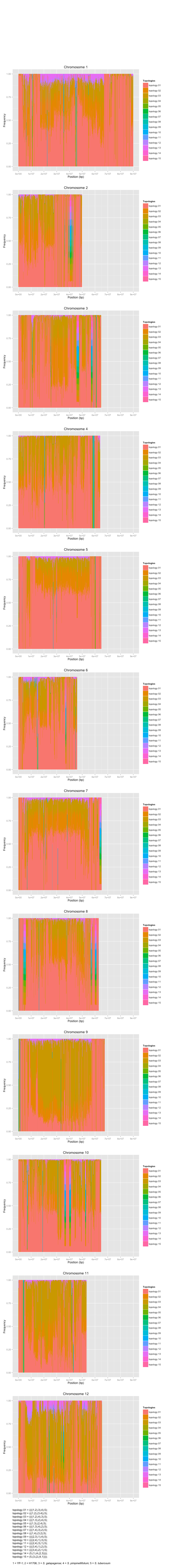

Supplement: Figure S3 [file peerj-03-793-s012.png]

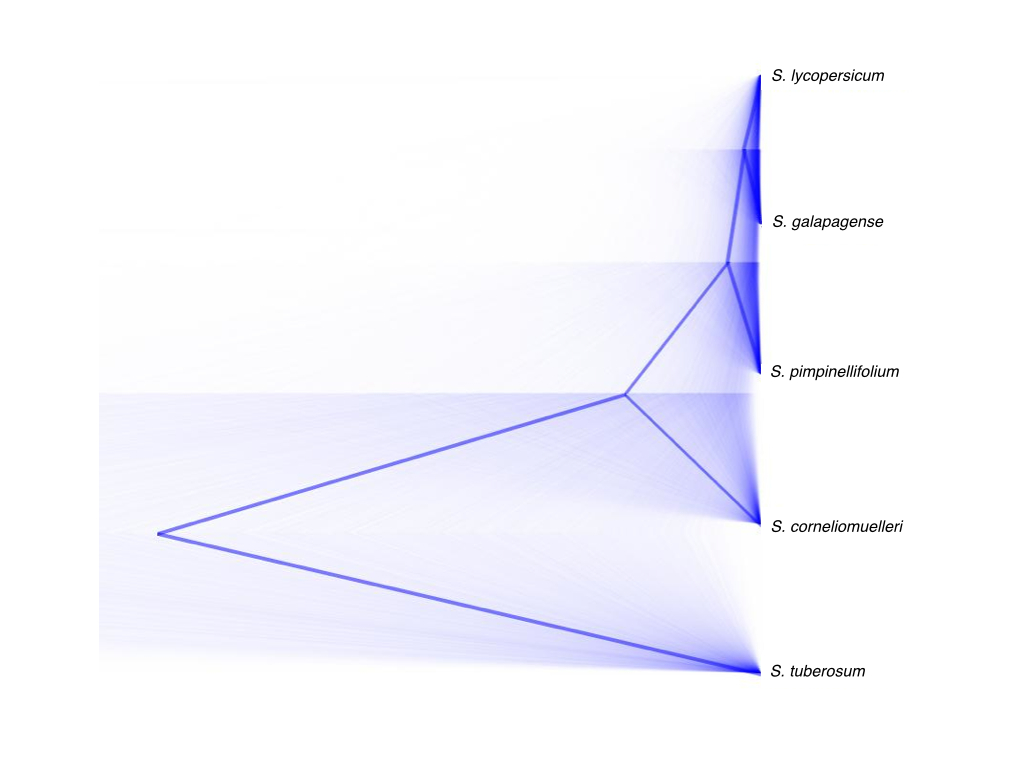

Supplement: File S4 [file peerj-03-793-s016.jpeg]

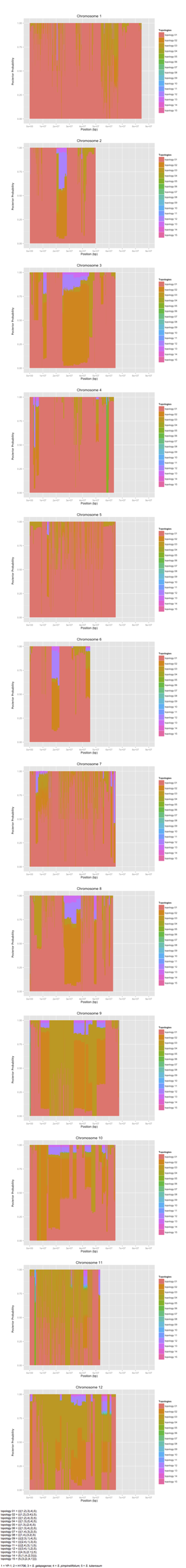

Supplement: File S5 [file peerj-03-793-s017.jpeg]
